# Novel insights into the genetic basis of buffalo reproductive performance

**DOI:** 10.1186/s12864-018-5208-6

**Published:** 2018-11-12

**Authors:** Jun Li, Jiajia Liu, Giuseppe Campanile, Graham Plastow, Chunyan Zhang, Zhiquan Wang, Martino Cassandro, Bianca Gasparrini, Angela Salzano, Guohua Hua, Aixin Liang, Liguo Yang

**Affiliations:** 1 College of Animal Science and Technology, Huazhong Agricultural University, Wuhan, Hubei China; 2 Department of Immunology, Zunyi Medical College, Zunyi, Guizhou China; 3 Department of Veterinary Medicine and Animal Productions, University of Naples “Federico II”, Naples, Italy; 4 Department of Agricultural, Food & Nutritional Science, University of Alberta, Edmonton, AB Canada; 5 Department of Agronomy, Food, Natural Resources, Animals and Environment, University of Padova, Agripolis, Legnaro, Italy

**Keywords:** Follicle, GWAS, Gene expression, *IGFBP7*, Reproduction

## Abstract

**Background:**

Fertility is a complex trait that has a major impact on the development of the buffalo industry. Genome-wide association study (GWAS) has increased the ability to detect genes influencing complex traits, and many important genes related to reproductive traits have been identified in ruminants. However, reproductive traits are influenced by many factors. The development of the follicle is one of the most important internal processes affecting fertility. Genes found by GWAS to be associated with follicular development may directly affect fertility. The present study combined GWAS and RNA-seq of follicular granulosa cells to identify important genes which may affect fertility in the buffalo.

**Results:**

The 90 K Affymetrix Axiom Buffalo SNP Array was used to identify the SNPs, genomic regions, and genes that were associated with reproductive traits. A total of 40 suggestive loci (related to 28 genes) were identified to be associated with six reproductive traits (first, second and third calving age, calving interval, the number of services per conception and open days). Interestingly, the mRNA expressions of 25 of these genes were also observed in buffalo follicular granulosa cells. The *IGFBP7* gene showed high level of expression during whole antral follicle growth. The knockdown of *IGFBP7* in buffalo granulosa cells promoted cell apoptosis and hindered cell proliferation, and increased the production of progesterone and estradiol. Furthermore, a notable signal was detected at 2.3–2.7 Mb on the equivalent of bovine chromosome 5 associated with age at second calving, calving interval, and open days.

**Conclusions:**

The genes associated with buffalo reproductive traits in this study may have effect on fertility by regulating of follicular growth. These results may have important implications for improving buffalo breeding programs through application of genomic information.

**Electronic supplementary material:**

The online version of this article (10.1186/s12864-018-5208-6) contains supplementary material, which is available to authorized users.

## Background

Buffalo is a mono-ovular species, during whose oestrus period, generally only one follicle matures and ovulates resulting in a major reduction in fertility, which restricts the development of the buffalo industry. Reproductive traits are quantitative traits influenced by many factors. With the application of genome-wide association studies (GWAS) for mapping genes for complex traits in domestic animals (e.g., pig, cattle and sheep), many important genes related to quantitative traits have been identified [1, 2]. However, only two GWAS have been performed for buffalo reproductive traits so far [3, 4]. In our previous study, nine significant (*P* < 0.001) SNPs associated with buffalo reproductive traits were identified by using the BovineSNP50 BeadChip from cattle [3]. With a significance threshold of *P* < 10^− 4^, 12 markers associated with four reproductive traits (age at first calving, calving interval, number of services per conception, and open days) of Murrah buffalo were detected by using a buffalo SNP chip array (Axiom ® Buffalo Genotyping Array 90 K Affymetrix) [4]. Mutations detected by GWAS were generally located in intergenic regions [5]. Whether these mutations affect target traits through specific pathways are unknown.

A thorough understanding of the biology of reproductive traits will be beneficial to the further development of buffalo breeding programs. Genetic variations are expected to alter gene expression leading to changes in the abundance of one or multiple proteins in turn affecting the phenotype of traits [6, 7]. Next generation sequencing (NGS) technology has been developed for the analysis of mRNA expression, and it has also been used for determining candidate genes which regulate reproductive performance of cattle [8] and buffalo [9]. Integrating GWAS and RNA-seq to detect important genes associated with target traits has been reported to provide new insight into the identity of causal genes and mutations and how they may influence the trait [10–13]. Up to now no reports related to buffalo reproductive traits with the integrated techniques have been available.

The manipulation of reproduction often relies on the regulation of follicle development during oestrous cycles. The process of folliculogenesis includes primordial follicle recruitment, followed by granulosa cell (GC) proliferation, and differentiation, and antral or preovulatory follicle formation [14, 15]. Every step of the folliculogenesis process is crucial for efficient fertility. In cattle, ovulation of large prolonged dominant follicles was reported to have resulted in a decrease in fertility [16], which was thought to be attributed to a reduction in fertilization rates or early embryo survival. Furthermore, oogenesis and folliculogenesis are inextricably linked, and the growth of oocytes are interdependent on that of the granulosa cells in the follicle [17]. The genes related to follicle development are therefore speculated to be important genes affecting the reproductive performance.

The 90 K commercial buffalo SNP chip, as an appropriate available tool [18], was applied to genotype 462 Italian Mediterranean buffaloes with 1424 lactations in this study. We therefore integrated GWAS results with our previous RNA-seq results [9] to identify the key genes affecting follicular development of buffalo. Our results lay the foundation for the understanding the genetic mechanism of buffalo reproduction.

## Results

### Genome-wide association study

Forty top suggestive (*P* < 10^− 4^) markers were observed (Table 1), 3 to 10 of which, were associated with the different traits. According to the cattle genome reference (*Bos taurus* UMD3.1 assembly), the SNPs of AX-85092311, AX-85128429, and AX-85136537 were found within genes (*TBCB, NDUFS2* and *TRHDE*). The remainders were aligned to intergenic regions (*SESN3, GPATCH4, IQGAP3, COL23A1, CADM2, FSTL4, BRINP2, KCNMA1, MEF2D, IGFBP7, NUFIP2, DIAPH3, CDH10, THRB, KYNU, CSGALNACT1, ZNF503, TRHDE, HYAL4, KRR1, MTPN, PELI2, SOX21, GMDS, PRDM5* and *LMO4*), which were spread over 17 chromosomes. Interestingly, the SNP of AX-85178307 was associated with TCA and SCA, and another SNP AX-85127260 was associated with TCA and CI, which is in line with the correlations observed between traits.

**Table 1:** Genes near to the most suggestive SNPs (*P* < 0.0001) for six reproductive traits

Traits	SNP ID	Chr	Position(bp)	Within Gene	Nearst Gene
FCA	AX-85104022	15	15,493,494	–	*SESN3*
	AX-85147160	15	15,464,584	–	*SESN3*
	AX-85066093	3	14,263,986	–	*GPATCH4*
SCA	AX-85115405	3	14,360,109	–	*IQGAP3*
	AX-85098201	7	40,737,269	–	*COL23A1*
	AX-85077363	1	31,982,649	–	*CADM2*
	AX-85041155	1	31,452,080	–	*CADM2*
	AX-85178307	7	46,586,723	–	*FSTL4*
TCA	AX-85178307	7	46,586,723	–	*FSTL4*
	AX-85097354	16	59,785,310	–	*BRINP2*
	AX-85109602	28	32,271,813	–	*KCNMA1*
	AX-85092311	18	46,969,939	*TBCB*	–
	AX-85115405	3	14,360,109	–	*MEF2D*
	AX-85160204	6	74,395,035	–	*IGFBP7*
	AX-85115163	19	21,212,867	–	*NUFIP2*
	AX-85128429	3	8,311,409	*NDUFS2*	–
	AX-85127260	28	32,296,913	–	*KCNMA1*
	AX-85060416	12	3,071,256	–	*DIAPH3*
NSC	AX-85122558	2	54,971,771	–	*KYNU*
	AX-85080363	20	48,102,049	–	*CDH10*
	AX-85149823	27	41,131,140	–	*THRB*
	AX-85080722	2	55,117,009	–	*KYNU*
	AX-85118378	27	37,743,905	–	*CSGALNACT1*
	AX-85069249	28	32,035,318	–	*ZNF503*
	AX-85069116	20	47,969,440	–	*CDH10*
OD	AX-85166811	5	2,459,053	–	*TRHDE*
	AX-85107057	5	2,430,266	–	*TRHDE*
	AX-85107924	5	2,493,145	–	*TRHDE*
	AX-85078259	5	2,531,719	–	*TRHDE*
	AX-85136537	5	2,378,071	*TRHDE*	–
	AX-85092056	5	2,670,570	–	*TRHDE*
	AX-85058327	4	89,251,931	–	*HYAL4*
	AX-85129886	5	5,220,162	–	*KRR1*
	AX-85080072	4	100,837,591	–	*MTPN*
CI	AX-85097797	10	69,031,255	–	*PELI2*
	AX-85056150	12	70,335,740	–	*SOX21*
	AX-85091977	23	51,462,571	–	*GMDS*
	AX-85082041	6	5,090,318	–	*PRDM5*
	AX-85127260	28	32,296,913	–	*KCNMA1*
	AX-85154020	3	56,690,373	–	*LMO4*

*FCA* Age at first calving, *SCA* Age at second calving, *TCA* Age at third calving, *CI* Calving interval, *NSC* The number of artificial insemination, *OD* The open days. Chr and Position were obtained based on the cattle genome (*Bos taurus* UMD3.1 assembly)

### Linkage disequilibrium and haplotype associations

**Fig. 1:**
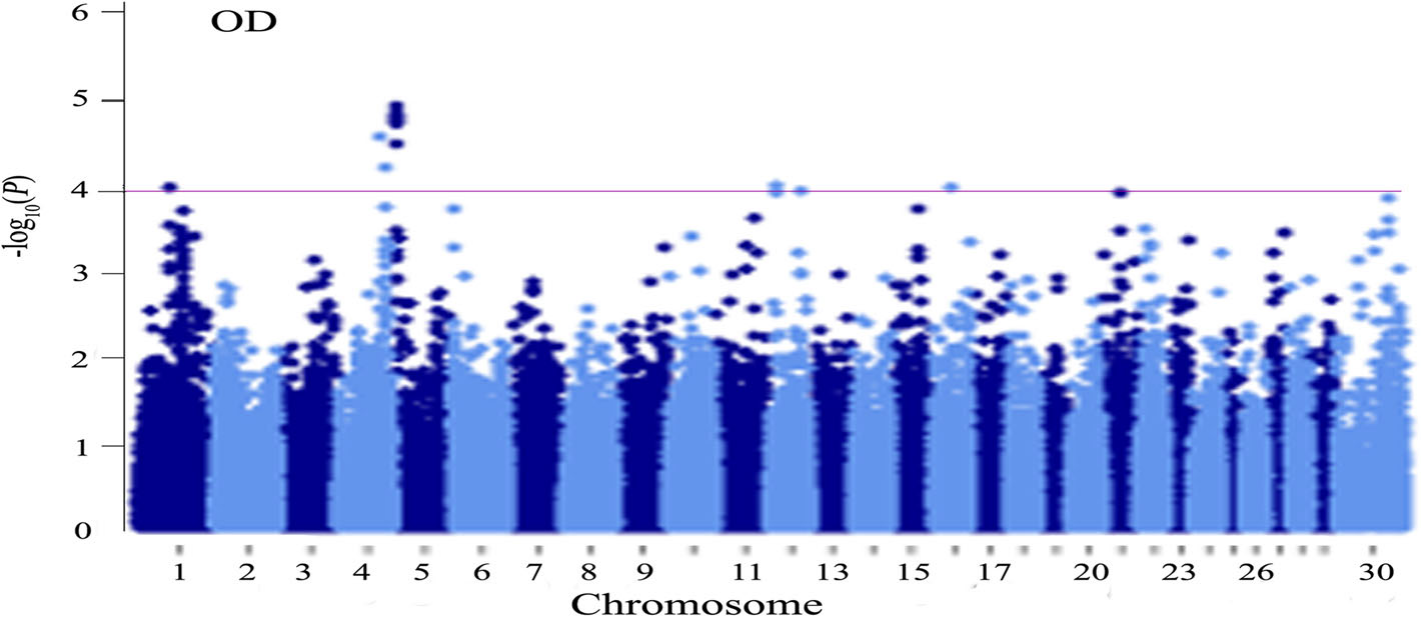
Manhattan plot for GWAS of open days in buffalo. OD, open days; x-axis, physical positions of SNPs by chromosome based on *Bos taurus* UMD3.1 genome assembly; y-axis, −log10 (*P*-values)

**Fig. 2:**
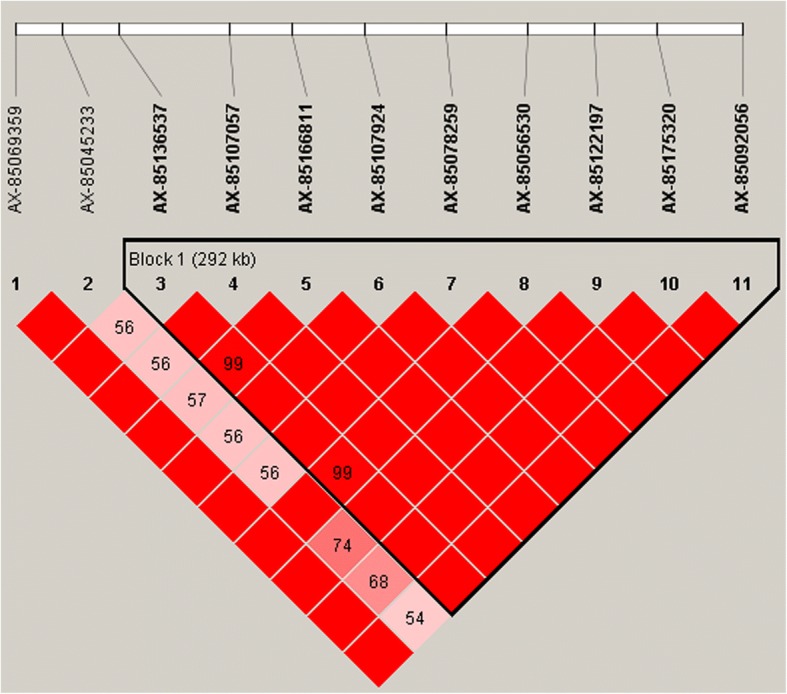
Linkage disequilibrium pattern for 11 SNPs, which have significant effects on OD by GWAS between 2 - 3Mbp on BTA5. Linkage disequilibrium block is presented in a triangle box based on LD (squared correlation coefficient, r2)

A particularly significant genomic region located at the equivalent 2.3–2.7 Mb of BTA5 (*Bos taurus* UMD 3.1 assembly), including six suggestive (*P* < 10^− 4^) SNPs, was identified to be associated with OD trait based on the GWAS results (Fig. 1). LD analysis indicated that a block (about 292 kb) was related to the above region (Fig. 2). Nine markers were in this block and they were in high LD, and most of them located in the *TRHDE* gene.

Four common haplotypes (CCGCATTAT, TTATGCCGC, TTATGCTAC, and CCGCACTAT) were inferred, and ten haplotype blocks were identified based on these four haplotypes. The analysis of relationship between haplotype blocks and buffalo reproductive traits revealed that haplotype blocks had significant effects on SCA (*P <* 0.05), CI (*P* < 0.001) and OD (*P* = 0.001). The multiple comparative analyses of the relationship between haplotype blocks and the three traits were shown in Additional file 1: Table S1, S2, and S3. The haplotype block (CCGCACTAT, TTATGCTAC) was associated with less second calving days, the haplotype block (CCGCATTAT, CCGCACTAT) was associated with the shortest calving interval, and the haplotype block (CCGCACTAT, CCGCACTAT) was associated with least open days.

### Potential genes identified by combining RNA-seq with GWAS

According to the RNA-seq results, 25 of 28 genes identified by GWAS were found to be expressed in buffalo follicular GCs (Table 2). The highest expression was found in *IGFBP7* during whole follicle growth in GCs, followed by *NDUFS2*, *TBCB*, *MTPN,* and *GMDS*. It is worth mentioning that the expression of the *CSGALNACT1* gene decreased gradually with the increase in follicular diameter, and was differentially expressed (FDR < 0.05) between small and large follicles (< 5 mm and > 8 mm in diameter).

**Table 2:** The expression level of genes associated with buffalo reproduction traits identified by GWAS during four stages of buffalo follicle growth

Gene	Length (bp)	RPKM*			
		< 5 mm	5~ 8 mm	8~ 12 mm	> 12 mm
*IGFBP7*	1107	723.49	840.17	965.37	883.67
*NDUFS2*	1601	215.46	254.71	317.72	322.20
*TBCB*	994	76.32	75.14	81.12	93.92
*MTPN*	2844	28.63	67.29	40.74	15.93
*GMDS*	1845	27.68	23.66	36.08	33.39
*ZNF503*	3514	21.09	20.43	25.47	15.84
*LMO4*	5219	15.44	31.11	35.43	23.79
*GPATCH4*	1830	10.96	8.31	8.36	9.72
*KRR1*	1258	7.67	20.06	9.86	4.39
*MEF2D*	5716	7.62	10.25	10.50	11.03
*DIAPH3*	4580	7.57	10.44	6.95	3.13
*NUFIP2*	3406	7.41	12.83	6.21	4.13
*KYNU*	2273	4.20	4.49	7.27	4.51
*CSGALNACT1* ^*#*^	3199	3.53^a^	0.87^ab^	0.42^b^	0.37^b^
*IQGAP3*	5860	3.51	4.93	3.85	1.71
*PRDM5*	6373	3.38	3.92	2.40	1.66
*PELI2*	5697	3.23	3.79	3.61	1.42
*SESN3*	7235	2.14	5.73	2.46	1.42
*THRB*	6346	1.89	1.65	2.55	2.12
*CADM2*	3342	0.03	0.17	0.04	–
*FSTL4*	8110	0.03	0.03	0.07	0.12
*KCNMA1*	2401	0.03	0.06	–	–
*COL23A1*	2333	–	–	0.04	0.03
*SOX21*	3417	–	0.02	–	–
*CDH10*	3174	–	0.09	–	0.07

*RPKM Reads per kb per million reads. ^#^CSGALNACT1: significanty differentially expressed gene (FDR < 0.05). The different superscript letters showed significant level

**Fig. 3:**
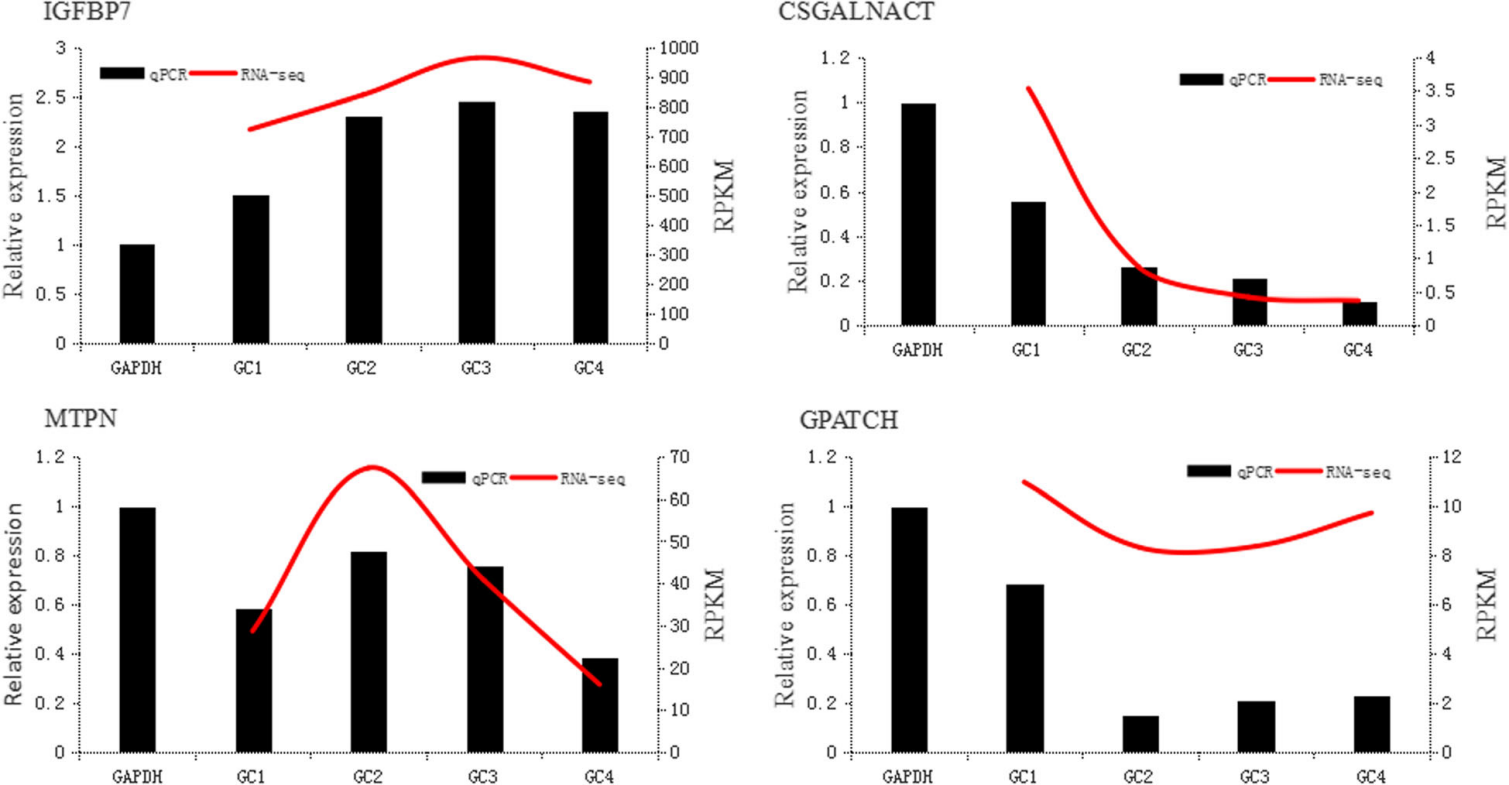
Expression of selected genes mRNA in buffalo granulosa cells detected by q-PCR compared with RNA-seq

Four genes (*IGFBP7*, *CSGALNACT*, *MTPN,* and *GPATCH*) with high expression level according to RNA-seq were selected for q-PCR validation. The expression patterns of these selected genes at four stages obtained by q-PCR were basically consistent with RPKM values obtained by RNA-seq (Fig. 3). *IGFBP7* was highly expressed during follicular development, so we chose this gene for subsequent functional verification.

### Knockdown of IGFBP7 affects cell growth

**Fig. 4:**
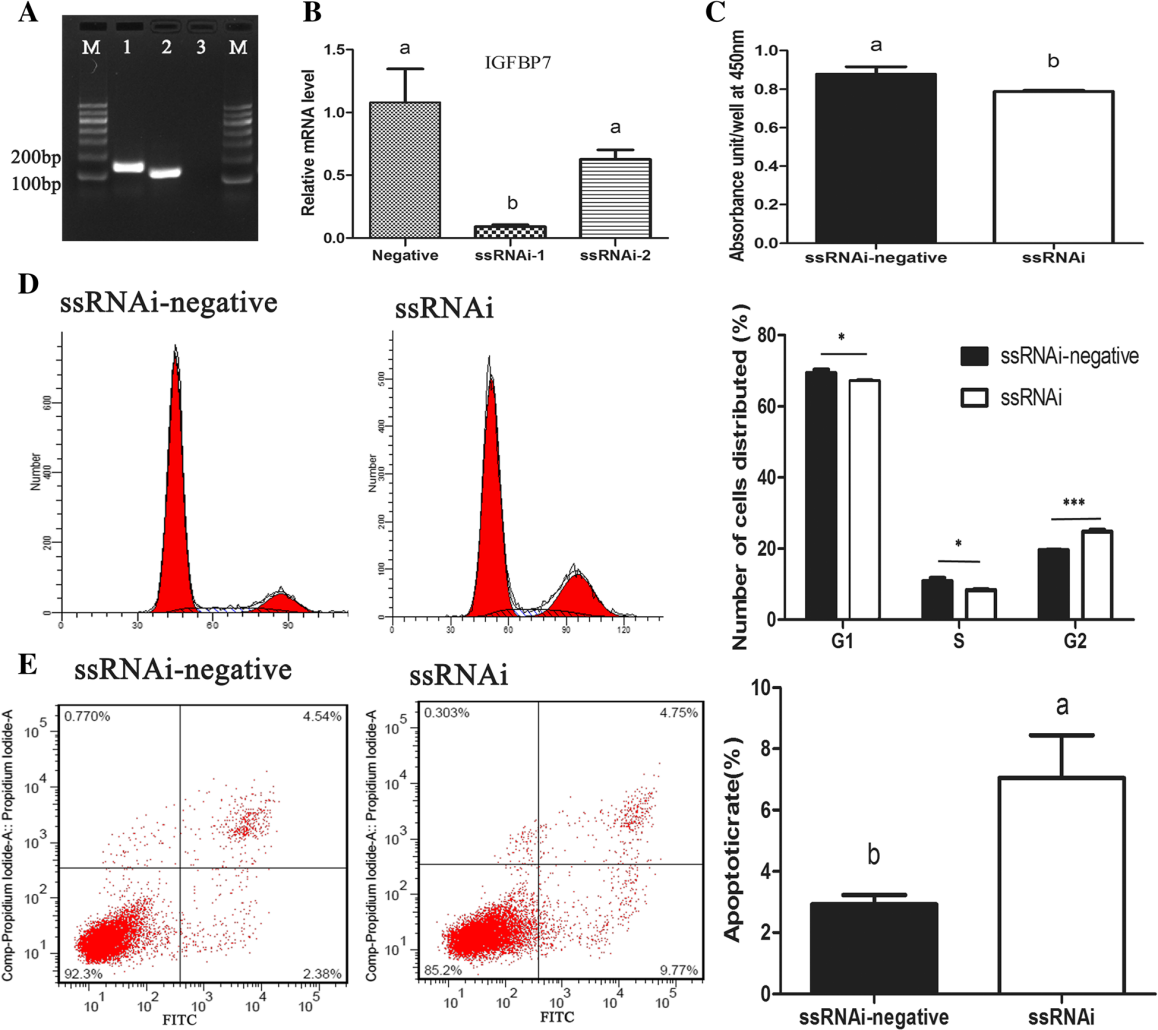
Apoptosis, proliferation and cycle analysis of buffalo granulosa cells (GCs) after transfection with IGFBP7 ssRNAi. **a** Expression of IGFBP7in buffalo GCs detected by reverse transcription polymerase chain reaction. Line 1 and 2 correspond to IGFBP7 and GADPH. **b** The mRNA expression level of IGFBP7 after transfection with IGFBP7 ssRNAi. **c** Analysis of buffalo GCs proliferation after knockdown of IGFBP7 with the absorbance at 450 nm. **d** and **e** showed analysis of cycles and apoptosis of buffalo granulosa cells after transfection with IGFBP7 ssRNAi, respectively. The values in each bar represents the mean ± SEM (*n* = 3). The different superscripts and the symbol of * on bars showed significant level (*P* < 0.05)

To investigate whether *IGFBP7* plays a role in the development of buffalo GCs, two recombinant RNAi vectors were constructed. Both RNAi vectors were effective in knocking down *IGFBP7* in buffalo GCs. The ssRNAi-1 vector had a better effect (98%) compared with ssRNAi-2 (Fig. 4b). Therefore, recombinant ssRNAi-1 vector was used for subsequent experiments.

**Fig. 5:**
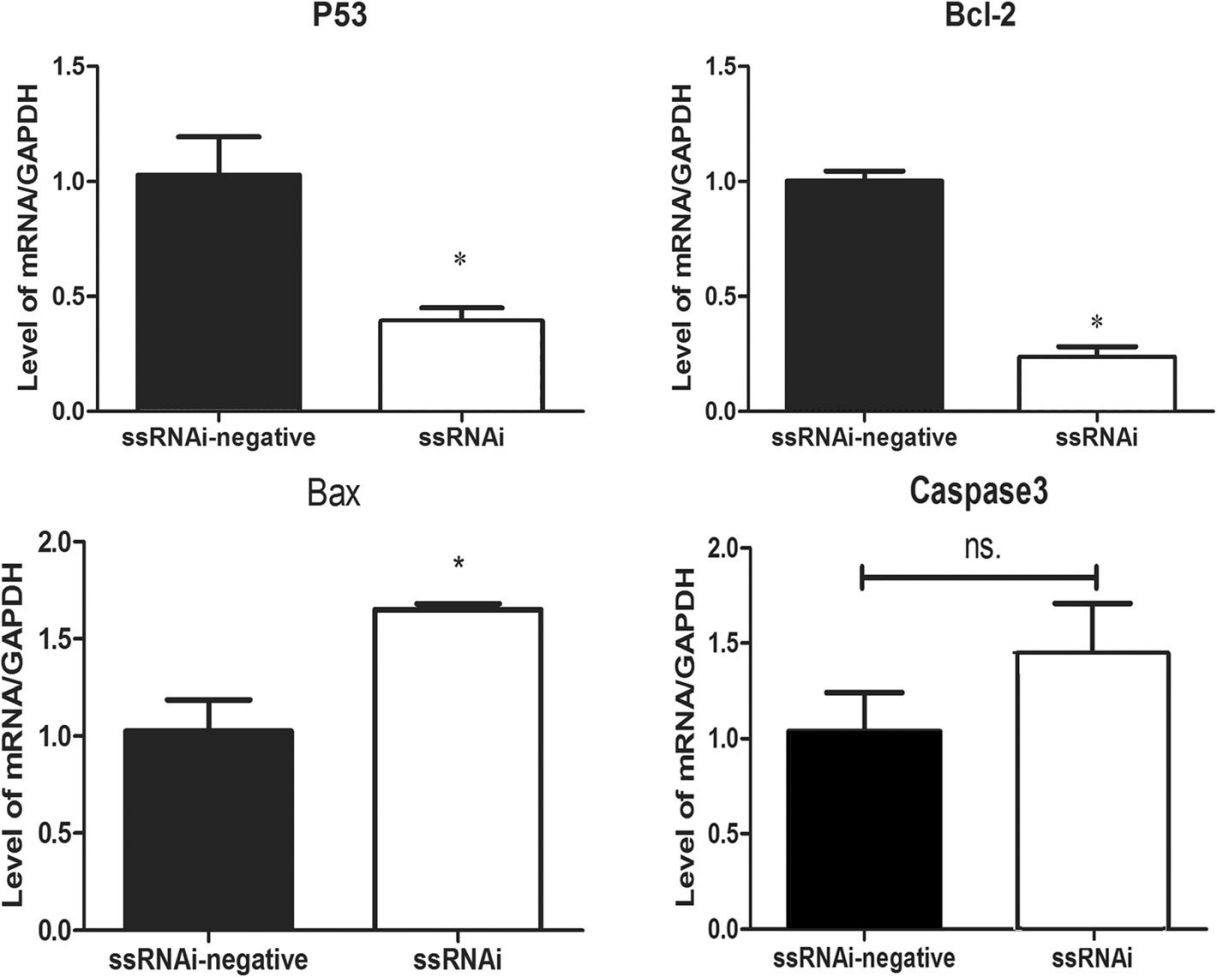
Effects on different apoptotic factors after knockdown of IGFBP7 in buffalo GCs. The mRNA expression of each apoptotic related genes was presented as mean ± SEM (*n* = 3). The symbol of * on bars showed significant level (*P* < 0.05). n.s. means none-significant

The knockdown of *IGFBP7* led to the arrest of the cell cycle and the increase in the proportion of early apoptotic cells. The percentage of GCs in G2 phase was significantly (*P* < 0.05) increased and the portions of GCs in the G1 and S phase were decreased (*P* < 0.05) (Fig. 4d). The *IGFBP7* silencing resulted in the significant increase in the number of apoptotic cells, compared with that of the control vector (7.5 ± 3.2 vs 2.9 ± 0.5, *P* = 0.003) (Fig. 4e). The results of apoptosis were also confirmed by detecting mRNA expression of *BCL2*, *BAX,* and *CASPASE3* after *IGFBP7* knockdown (Fig. 5). The knockdown of *IGFBP7* also suppressed buffalo GCs proliferation. The absorbance at 450 nm in ssRNAi transfected cells was higher than that of control group (Fig. 4c).

### Knockdown of IGFBP7 increased basal progesterone and E2 production

**Fig. 6:**
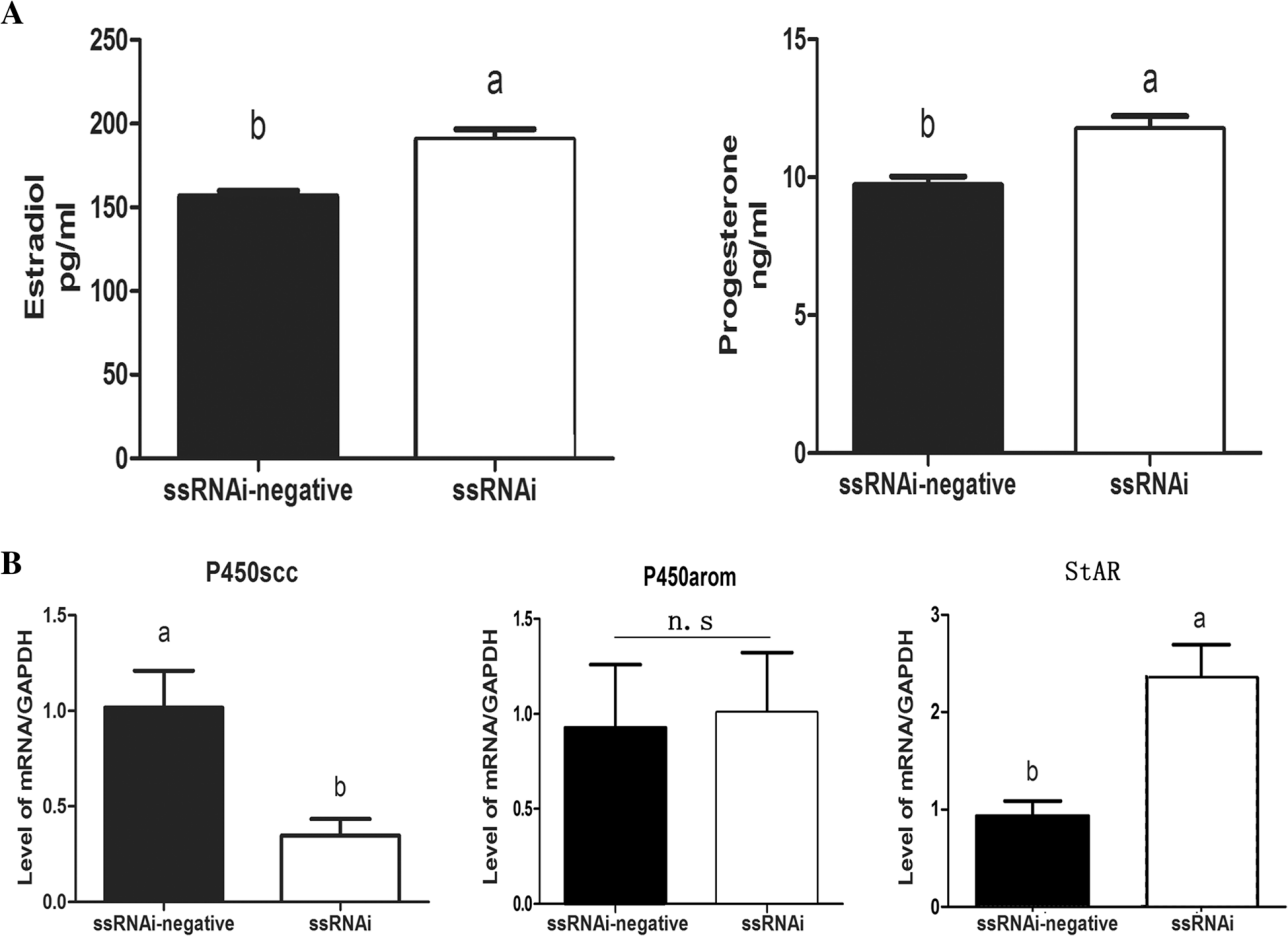
Production of progesterone and E2 and the expression level of related genes after knockdown of IGFBP7 in buffalo GCs. **a** Detection of progesterone and E2 secretion by ELISA Kit. **b** Analysis of mRNA expression level of hormone related genes. The values in each bar represents the mean ± SEM (*n* = 3). The different superscripts on bars showed significant level (*P* < 0.05). n.s. means none-significant

The production of progesterone and E2 increased (*P* < 0.05) by knockdown of *IGFBP7* in buffalo GCs cultured for 48 h after transfection (Fig. 6a). Furthermore, *IGFBP7* silencing increased the mRNA expression of *STAR* and decreased the mRNA expression of *CYP11A1* while no significant change in mRNA expression of *CYP21A2* was observed (Fig. 6b).

## Discussion

In a previous study, 12 significant (*P* < 10^− 4^) SNPs associated with four reproductive traits were identified by GWAS in Murrah buffalo [4]. Nonetheless, these 12 significantly associated SNPs were not detected in the present study. Furthermore, a recent study showed more than 150 SNPs were associated with indicator traits of sexual precocity [19], however, none of them are identified in this study. Many reports indicated that GWAS has some limitations [20, 21], therefore, the functional verification analysis of significant SNPs detected by GWAS remains to be conducted. In a previous study, integrating sequence-based GWAS and RNA-seq provided novel insights into the genetic basis of mastitis and milk production in dairy cattle [10]. In the present study, we used GWAS to detect key genes associated with buffalo reproductive traits, and the results of RNA-seq were used to explore the changes in the expression of these key genes during the development of follicles. The expression of 25 out of 28 genes associated with buffalo reproductive traits was also detected in buffalo follicular GCs by RNA-seq, indicating that these genes may have affected the fertility by being directly through the regulation of buffalo follicle growth.

Hormones play an essential role in animal reproductive performance, and hormones and antibiotics have been applied as treatments in animal reproduction studies [22]. In the present study, five notable genes related to hormonal regulation were identified: *TRHDE* [23] for thyrotropin, *KCNMA1* [24], *TBCB* [25] and *CDH10* [26] for prostrate hormones, and *THRB* [27] for thyroid hormones. Thyroid dysfunction is associated with anovulation, abortion, and infertility in women and experimental animals [28]. Prostaglandin concentration is abnormally high in human ejaculates, and the prostate has specific dependence on androgen stimulation [29]. Thyroid hormones play a key role in seasonal cycles of body weight and reproduction [30]. Coincidentally, buffalo is also a seasonal estrus species. Furthermore, the mutation of *THRB* was reported to be significantly associated with thyroid hormone resistance (*THR*) [27]. *KCNMA1* gene can enhance erectile strength and sexual behavior [31]. *TRHDE* encoding a thyrotropin releasing enzyme was reported to be associated with a variety of phenotypes including growth traits in Ujumqin sheep [23], the adaptation of goats to hot arid environments [32] and rheumatoid arthritis [33] in GWAS studies. According to the RNA-seq results, the expression of these hormone-related genes is low in buffalo granulosa cells, but we cannot ignore the impact of these genes because low levels of hormone may be highly effective in the regulation of follicle growth [34]. It is reasonable to assume that genes involved in hormonal regulation could be associated with buffalo fertility, and that these markers may have high utilization value for buffalo breeding.

Buffalo reproductive traits are controlled by polygenes, and many genes of small effects can produce synergistic effects on a pathway. Function based analysis can be used to detect the combined effects of these genes. In the present study, five important genes, *NDUFS2*, *GMDS*, *KYNU*, *HYAL4,* and *CSGALNACT1*, were classified into metabolic pathways. Metabolism is one of the most basic processes in the body, and plays a crucial role in ensuring the normal performance of various life activities. These genes may affect the reproductive performance of buffalo by regulating glucose, lipid, protein, and nucleic acid metabolism. Extensive information has previously been obtained about genes involved in metabolic pathways affecting animal fertility [35]. Among these genes, the *CSGALNACT1* gene, which is required for normal cartilage development [36], was found to be significantly down-regulated by comparing buffalo follicles with diameter < 5 mm and > 8 mm in the present study. Based on it, we hypothesize that *CSGALNACT1* gene has effect on the growth of buffalo follicles by changing the level of expression to regulate glucose metabolism.

In our previous study, *CADM2* (cell adhesion molecule 2) was found to be associated with buffalo reproductive performance by using the BovineSNP50 BeadChip [3]. One SNP located at about 143 kb of the upstream bovine *CADM2* was associated with days from first calving to conception and calving interval in days from first to second calving. In the present study, another SNP AX-85077363 located downstream of the bovine *CADM2* gene was identified to be associated with SCA trait. However, the expression of *CADM2* was only detected in early follicular GCs. We speculate that the two sites nearby the *CADM2* gene may interact with other significant SNPs or genes to influence buffalo reproductive performance.

*ZNF503*, *KRR1,* and *MTPN* are other target genes associated with buffalo reproductive traits identified in this study. *ZNF503*, also known as *Nolz-1*, which promotes mammary epithelial cell proliferation and enhances cell invasion [37], plays an important role in embryogenesis [38]. *KRR1* was reported to be associated with polycystic ovary syndrome (PCOS) in European cohorts [39]. *MTPN* plays an essential role in cell and skeletal muscle growth [40], and it is related to antigen recognition which is a key process in immune response [41]. These three genes were highly expressed in buffalo GCs with follicular diameter 5 ~ 12 mm, suggesting that they may have participated in dominant follicle selection.

*IGFBP7* shares sequence homology with follistatin [42] which is originally considered as an inhibitor of FSH secretion [43, 44] and plays a crucial role in folliculogenesis and ovarian function [45, 46]. The expression of *IGFBP7* was also identified in the GCs of the large antral follicles of porcine ovary [47] and bovine corpus luteum [48]. Therefore, we speculate that *IGFBP7* may participate in the regulation of follicle development and ovulation. In the present study, our RNA-seq results showed that *IGFBP7* is highly expressed in GCs of buffalo antral follicles. Moreover, the knockdown of *IGFBP7* in buffalo GCs affected the number of apoptotic cells, cell cycles, cell proliferation, and the production of estrogen and progesterone. These observations indicate that *IGFBP7* may be involved in the regulation of follicle development.

## Conclusions

This is the first attempt combining GWAS and RNA-seq to explore genetic mechanism of buffalo reproductive performance. A total of 25 important genes were identified to be associated with buffalo reproductive performance, and they may affect the buffalo reproductive traits through the regulation of follicle development. The study expands the knowledge of the genetic basis of buffalo fertility, and it may also benefit future research on improving buffalo breeding programs.

## Materials and methods

### Animal and data collection

The data and samples used for GWAS were obtained from the Italian Buffalo Breeders Association, which is responsible for the buffalo production program in Italy.

The data analysis was based on 1424 lactations of 462 Italian Mediterranean buffaloes born in 2000–2011 in four Italian farms. Six reproductive traits were examined including first calving age (FCA), second calving age (SCA), third calving age (TCA), calving interval (CI), number of services per conception (NSC), and open days (OD).

The trait FCA is defined as the days from the birth to first calving of the buffalo; SCA is defined as the days from birth to second calving of the buffalo; and the days between the third calving and the birth of buffalo is defined as TCA. The CI is defined as the days between consecutive events. The trait NSC refers to the times of artificial insemination (AI) that is required for each buffalo to conceive. The OD is defined as the days from calving to the next conception.

Data structure is important as they underpin GWAS results. The descriptive statistics of phenotypes of buffalo reproductive traits are presented in Table 3.

**Table 3:** Descriptive statistics of studied reproduction traits

Traits	Records	Mean	SD	Max	Min
FCA	483	1015.44	157.08	1769	574
SCA	398	1491.70	208.64	2317	797
TCA	230	1908.86	255.79	3071	1185
CI	891	431.55	75.67	657	235
NSC	1110	2.27	0.68	6	1
OD	1008	120.04	61.58	270	40

*SD* Standard deviation, *FCA* Age at first calving, *SCA* Age at second calving, *TCA* Age at third calving, *CI* Calving interval, *NSC* The number of artificial insemination, *OD* Open days

### Genotyping and quality control

Genomic DNA was isolated from the blood leucocytes of Italian buffalo using a standard phenol-chloroform extraction protocol and the DNA was genotyped using the 90 K Axiom Buffalo SNP Array at Delta Genomics (Edmonton AB, Canada). Sample quality control used animal call rates > 97%. For single nucleotide polymorphisms (SNPs), thresholds were set as SNPs call rates > 95%, minor allele frequency (MAF) > 0.05 and Hardy - Weinberg equilibrium (HWE) test *P*-value < 10^− 6^. Unmapped SNPs were eliminated and markers present on the Y chromosome were removed. After quality control, 62,747 SNPs and 462 buffaloes were used for subsequent analyses.

### Genome wide association study

The association study was performed using a ridge regression BLUP (rrBLUP) model in both PLINK and R software.

$$ y= X\beta +u+e $$

Where ***y*** is the vector of reproductive traits, ***X*** is a vector of coded SNP genotypes, which is assigned to − 1, 0, or 1 for genotypes AA, AB, and BB, respectively. ***Β*** is the SNP effect; ***u*** is the vector of the polygenic effects, and ***e*** is the vector of random residuals. ***u*** and ***e*** are assumed to be subject to the normal distribution, ***[u ~(0,Kσ***_***u***_^***2***^***)]*** and ***[e ~ (0, Iσ***_***e***_^***2***^***)]***, where ***K*** is the genomic relationship matrix based on pedigree information, ***σ***_***u***_^***2***^ is the genetic variance, ***I*** is an identify matrix, ***σ***_***e***_^***2***^ is the residual variance. The number of potential markers was too small after Bonferroni correction (0.05/62747 = 7.97 × 10^− 7^), resulting in very low statistical power. Therefore, we used suggestive *P* value (*P* < 0.0001) as the threshold in this analysis.

### Haplotype analyses

The linkage disequilibrium (LD) between markers was calculated as squared correlation coefficient (r^2^) of alleles using Haploview 4.2 software. The distances of pairwise SNPs less than 500 kb were used to draw the LD figure. Haplotype blocks were constructed by using PHASE software, and the association between each haplotype block and six reproductive traits were estimated with Bonferroni t test in R. We used suggestive *P* value (*P* < 0.05) as threshold for considering candidate haplotype blocks affecting buffalo reproductive traits.

### Identification of SNP location

Two available buffalo genome sequences on the NCBI platform are not displayed on chromosomes and genes are not annotated (UMD CASPUR WB 2.0; https://www.ncbi.nlm.nih.gov/assembly/GCF_000471725.1/). Cattle and buffalo have been reported to be closely related, sharing homology in chromosome banding and gene mapping [49, 50]. It has also been confirmed that the bovine genome is a useful source of markers for buffalo genome mapping [51, 52], three available buffalo GWAS were constructed using the cattle genome reference [4, 18, 53]. In the present study, the SNP location was identified using the cattle genome sequence.

### Gene expression in buffalo follicular GCs

The normal development of follicles directly affects buffalo reproductive performance. We speculated that key genes associated with reproductive performance identified by GWAS may be involved in the regulation of follicular development. Therefore, we integrated GWAS results with our previous RNA-seq results [9] to explore the expression of key genes in buffalo GCs during follicular development. The expressions of significant genes identified by GWAS were obtained by RNA-seq.

### Quantitative real-time polymerase chain reaction (q-PCR) validation

Ovaries were collected from a local abattoir in Wuhan, China, from nonpregnant buffaloes. The ovaries with lutealized and large cystic follicles were detected and discarded. The largest follicles from normal ovaries were collected. Follicles were graded into 4 categories according to their diameters, namely, diameter < 5 mm, 5 ~ 8 mm, 8 ~ 12 mm and > 12 mm, and morphological appearance was used to assess the developmental stages of the follicles. GCs were collected from the follicles after grading and each category included 10 follicles. Single-stranded cDNA was obtained from 1 μg total RNA isolated from each of the groups. Primers (Additional file 1: Table S4) for q-PCR were designed using Primer Premier 5.0 software and synthesized by Sangon Biotech (Shanhai) Co. Ltd. q-PCR was performed on LightCycler 480II (Roche), using a SYBR Green-based PCR assay. A reaction system consists of a small aliquot of the cDNA (100 ng), 10 μL PCR master mix (TaKaRa, Japan), 2 μL of primers (1 μL of each forward and reverse primer, 10 pmol/μL), and 7 μL ddH_2_O. The q-PCR was run as follows: 50 °C for 2 min, 95 °C for 10 min, followed by 35 cycles of 95 °C for 30 s, annealing temperatures for 30 s (the annealing temperature for each gene was shown in Additional file 1: Table S4), and 72 °C for 30 s in 96-well optical reaction plate. The expression levels of the tested genes were determined by CT values and the final expression for each gene was normalized against the value of *GAPDH*. The analyses were conducted with three biological replicates.

*Functional verification by RNA interference technique.*

Isolation and culture of buffalo granulosa cells were described in our previous study [54]. Two ssRNAi vectors (Additional file 1: Table S5) of insulin-like growth factor-binding protein 7 (*IGFBP7*) were constructed according to the coding sequence (Accession number: XM_006058372). These two plasmids and another negative control plasmid were named ssRNAi-1, ssRNAi-2, and ssRNAi-negative, respectively. The method of transfection was described in our previous study [54].

After transfection with plasmid, the mRNA expression of *IGFBP7*, apoptosis related genes, and hormone synthesis related genes were detected by q-PCR which was described above. AnnexinV-FITC/PI kit (KGA107, Kaiji Biotechnology Co., Ltd., Nanjing, China) was used to measure the apoptosis of GCs according to the manufacturer’s protocol. Cell cycle detection was also performed using flow cytometry and the excitation wavelength was 488 nm according to the manufacturer’s protocol of cell cycle detection kit (KGA512, Kaiji Biotechnology Co., Ltd., Nanjing, China). The analysis of cell proliferation was performed using the Cell Counting Kit (catalogue number: ck04) with absorbance wavelength being 450 nm. Moreover, the cell culture medium was collected and the production of estrogen and progesterone was measured using a Bovine ELISA Kit (catalogue number: JYM0045Bo and JYM0056Bo, respectively) according to the manufacturer’s instructions.

## Additional file

Additional file 1: **Table S1.** Multiple Comparative Analysis of the relationship between Buffalo Haplotype block and SCA Trait. **Table S2.** Multiple Comparative Analysis of the relationship between Buffalo Haplotype block and CI Trait. **Table S3.** Multiple Comparative Analysis of the relationship between Buffalo Haplotype block and OD Trait. **Table S4.** Primers used for quantitative real-time polymerase chain reaction. **Table S5.** siRNA description. (DOC 64 kb)

## Abbreviations

CI: Calving interval
FCA: First calving age
GC: Granulosa cell
GWAS: Genome-wide association study
HWE: Hardy - Weinberg equilibrium
LD: Linkage disequilibrium
MAF: Minor allele frequency
NGS: Next generation sequencing
NSC: Number of services per conception
OD: Open days
q-PCR: Quantitative real-time polymerase chain reaction
SCA: Second calving age
SNP: Single nucleotide polymorphism
TCA: Third calving age
