# Supplementary material for: Novel insights into the genetic basis of buffalo reproductive performance
Source: BMC Genomics. 2018 Nov 12;19:814. doi: 10.1186/s12864-018-5208-6 (PMC6233259; doi:10.1186/s12864-018-5208-6)
Supplement: Supplementary file 1 — Table S1. Multiple Comparative Analysis of the relationship between Buffalo Haplotype block and SCA Trait. Table S2. Multiple Comparative Analysis of the relationship between Buffalo Haplotype block and CI Trait. Table S3. Multiple Comparative Analysis of the relationship between Buffalo Haplotype block and OD Trait. Table S4. Primers used for quantitative real-time polymerase chain reaction. Table S5. siRNA description. (DOC 64 kb) [file 12864_2018_5208_MOESM1_ESM.doc]

**Additional file 1: Table S1** Multiple Comparative Analysis of the relationship between Buffalo Haplotype block and SCA Trait

| SCA | Records | Mean | SD | Min | Max |
| --- | --- | --- | --- | --- | --- |
| TTATGCCGC,TTATGCCGC | 25 | 1566.84^a^ | 211.925 | 1148 | 1956 |
| CCGCATTAT,TTATGCCGC | 59 | 1548.47^a^ | 207.866 | 1151 | 2257 |
| TTATGCTAC,TTATGCCGC | 29 | 1524.90^ab^ | 235.130 | 1184 | 2167 |
| CCGCACTAT,TTATGCCGC | 16 | 1515.69^abc^ | 174.752 | 1301 | 1871 |
| CCGCATTAT,CCGCATTAT | 69 | 1502.87^abc^ | 237.087 | 797 | 2317 |
| CCGCATTAT,TTATGCTAC | 38 | 1478.89^abc^ | 218.420 | 1177 | 2180 |
| TTATGCTAC,TTATGCTAC | 15 | 1449.00^abc^ | 266.744 | 1062 | 2041 |
| CCGCATTAT,CCGCACTAT | 45 | 1422.89^bc^ | 192.726 | 1172 | 1974 |
| CCGCACTAT,CCGCACTAT | 10 | 1388.10^bc^ | 209.888 | 1072 | 1810 |
| CCGCACTAT,TTATGCTAC | 16 | 1387.56^c^ | 198.844 | 843 | 1639 |

SCA, age at second calving; SD, standard deviation

The different letters on the shoulders show the significant differences on the same column (*P* < 0.05).

**Additional file 1: Table S2** Multiple Comparative Analysis of the relationship between Buffalo Haplotype block and CI Trait

| CI | Records | Mean | SD | Min | Max |
| --- | --- | --- | --- | --- | --- |
| TTATGCTAC,TTATGCTAC | 42 | 487.19^a^ | 132.220 | 325 | 889 |
| TTATGCTAC,TTATGCCGC | 52 | 470.46^a^ | 110.589 | 337 | 759 |
| TTATGCCGC,TTATGCCGC | 71 | 466.01^ab^ | 109.190 | 346 | 916 |
| CCGCATTAT,CCGCATTAT | 149 | 457.62^abc^ | 132.949 | 82 | 1316 |
| CCGCATTAT,TTATGCCGC | 139 | 456.94^abc^ | 117.553 | 336 | 1260 |
| CCGCATTAT,TTATGCTAC | 73 | 431.81^bcd^ | 74.730 | 338 | 696 |
| CCGCACTAT,TTATGCCGC | 40 | 426.83^bcd^ | 74.517 | 347 | 715 |
| CCGCACTAT,TTATGCTAC | 26 | 423.12^bcd^ | 124.332 | 340 | 907 |
| CCGCACTAT,CCGCACTAT | 36 | 422.00^cd^ | 93.103 | 336 | 745 |
| CCGCATTAT,CCGCACTAT | 124 | 413.74^d^ | 74.669 | 325 | 700 |

CI, calving interval; SD, standard deviation

The different letters on the shoulders show the significant differences on the same column (*P* < 0.05).

**Additional file 1: Table S3** Multiple Comparative Analysis of the relationship between Buffalo Haplotype block and OD Trait

| OD | Records | Mean | SD | Min | Max |
| --- | --- | --- | --- | --- | --- |
| TTATGCCGC,TTATGCCGC | 75 | 146.21^a^ | 75.126 | 34 | 363 |
| TTATGCTAC,TTATGCTAC | 50 | 145.56^ab^ | 81.451 | 36 | 333 |
| TTATGCTAC,TTATGCCGC | 62 | 138.10^abc^ | 75.912 | 25 | 328 |
| CCGCATTAT,TTATGCCGC | 160 | 127.24^abc^ | 72.485 | 24 | 304 |
| CCGCATTAT,CCGCATTAT | 175 | 125.00^bc^ | 79.738 | 20 | 418 |
| CCGCATTAT,TTATGCTAC | 102 | 115.78^cd^ | 64.716 | 26 | 316 |
| CCGCACTAT,TTATGCCGC | 47 | 114.57^cd^ | 58.694 | 35 | 236 |
| CCGCACTAT,TTATGCTAC | 43 | 111.19^cd^ | 75.570 | 31 | 413 |
| CCGCATTAT,CCGCACTAT | 149 | 104.72^d^ | 62.159 | 26 | 295 |
| CCGCACTAT,CCGCACTAT | 37 | 101.11^d^ | 50.740 | 24 | 260 |

OD, the open days; SD, standard deviation

The different letters on the shoulders show the significant differences on the same column (*P* < 0.05).

**Additional file 1: Table S4** Primers used for quantitative real-time polymerase chain reaction

| Gene | Primer sequence (5’-3’) | Length of product (bp) | Tm (◦C) |
| --- | --- | --- | --- |
| [*β-Actin*](http://www.baidu.com/link?url=qtcwGJnon2MkZtTn4xs0dAjX6Yk_-iK3U4heTecnn4_rqNfNwDMEMPj92bPsrPtZIjuhzCnnXw4X3o_ejgIafz3ZJjmjRmnNv16GUAG1SYi) | F: GCCCTGGCACCCAGCACAAT  R:GGAGGGGCCGGACTCATCGT | 300 | 56 |
| *IGFBP7* | F: AGCAAGGTCCTTCCATCGTG  R: AGCACCCAGCCAGTAACTTC | 232 | 58 |
| *CSGALNACT1* | F: TCACGATGCAATCCCTGCTT  R: TGATGTCCAGGTCGAAACCG | 137 | 58 |
| *MTPN* | F: CATGTGGGCCCTGAAAAACG  R: AAAGGCAGTCAGTCCATCGG | 298 | 57 |
| *GPATCH4* | F: ACTCGACAGGACGGAGTACA  R: AGGCCTGGAGTAGCATCTCA | 217 | 59 |
| *P53* | F: AGACATTTTCCGACTTGTGG  R: GCACTTCATTCGGACATTCA | 132 | 59 |
| *BCL2* | F: ATGTGTGTGGAGAGCGTCAA  R: CTAGGGCCATACAGCTCCAC | 146 | 57 |
| *BAX* | F: CCTTTTGCTTCAGGGTTTCA  R: CGCTTCAGACACTCGCTCA | 300 | 56 |
| *CASPASE3* | F: AAGCCATGGTGAAGAAGGAA  R: GGCAGGCCTGAATAATGAAA | 134 | 58 |
| *CYP11A1* | F: AGACTTGGAGGGACCATGTAG  R: TGTCATGGATGTCGTGTCCA | 218 | 59 |
| *CYP21A2* | F: CCTCAATACCAGGTCCCAGC  R: TCTCGTGCATGCCGATGAAT | 255 | 57 |
| *STAR* | F: TCAGCTGGAAGACCCTCTCT  R: CAGCCCTCCTGGTCTTTGAG | 105 | 58 |

**Additional file 1: Table S5** siRNA description.

| SiRNA | Sequence (5’–3’) |
| --- | --- |
| ssRNAi-1 | GCAAGAGGCGGAAGGGUAAT |
| ssRNAi-2 | CCAUGCAUCCAAUUCCCAATT |
| ssRNAi-negative | UUCUCCGAACGUGUCACGUTT |
